# Colorectal cancer mortality is associated with low selenoprotein P status at diagnosis

**DOI:** 10.1016/j.redox.2025.103701

**Published:** 2025-05-24

**Authors:** Stefanie Brezina, Thilo Samson Chillon, Sabrina Asaad, Andreas Maieron, Julian Prosenz, Julian Seelig, Kamil Demircan, David J. Hughes, Andrea Gsur, Lutz Schomburg

**Affiliations:** a Center for Cancer Research, Medical University of Vienna, Borschkegasse 8a, 1090, Vienna, Austria; b Institute for Experimental Endocrinology, Charité-Universitätsmedizin Berlin, Hessische Straße 4A, 10115, Berlin, Germany; c Department of Clinical Biochemistry, Odense University Hospital, 5000, Odense, Denmark; d Department of Internal Medicine 2, University Hospital-St. Pölten, Karl Landsteiner University of Health Sciences, 3100, St. Pölten, Austria; e Klinik und Poliklinik für Onkologie, Gastroenterologie, Hepatologie und Pneumologie, Liebigstr. 20, 04103, Leipzig, Germany; f Precision Healthcare University Research Institute, Queen Mary University of London, London, UK; g Computational Medicine, Berlin Institute of Health at Charité-Universitätsmedizin Berlin, Germany; h Molecular Epidemiology of Cancer Group, School of Biomolecular and Biomedical Science, UCD Conway Institute, University College Dublin, Dublin, Ireland

**Keywords:** Selenium, Trace element, Glutathione peroxidase, Mortality risk, Prediction

## Abstract

Selenium (Se) deficiency, affecting hundreds of millions of individuals worldwide, is linked to increased incidence of colorectal cancer (CRC), yet tumors paradoxically accumulate Se to evade ferroptosis and promote metastasis. Therefore, understanding the prognostic impact of Se status at diagnosis is crucial to consider and enable personalized interventions.

Four Se markers, namely total-Se, the circulating selenoproteins GPx3 and SELENOP, and autoantibodies to SELENOP, were analyzed in participants of the ongoing Colorectal Cancer Study of Austria (CORSA). Final analyses included 519 participants (n = 153 tumor-free, n = 255 adenoma and n = 111 CRC). Subjects were enrolled following a positive fecal immunochemical test, underwent a colonoscopy for diagnosis, and were followed up for 15 years.

Total-Se concentration and GPx3 activity did not differ across groups, but SELENOP concentrations were lower in CRC (median (IQR); controls: 2.9 (0.9), adenoma: 2.8 (1.0), CRC: 2.4 (0.9); p < 0.001). Prevalence of SELENOP autoimmunity was <1 % in controls, but >5 % in patients. Total Se and SELENOP levels above the median were associated with better survival in all groups. SELENOP displayed an inverse association with mortality in fully adjusted models (HR(CI) per SD for SELENOP; controls: 0.62(0.46–0.83), adenomas: 0.73(0.59–0.90), CRC: 0.64(0.49–0.84)). Adding any Se biomarker, particularly SELENOP, to a model with established clinical parameters improved prognostication, and the highest prognostic values were observed when including SELENOP or all three Se biomarkers. Data-driven clustering analysis identified three distinct clusters based on Se markers, one of which displayed a remarkably increased risk for mortality (HR; 1.8).

We conclude that SELENOP deficiency at the time of diagnosis is inversely associated with mortality risk and improves prognostication over clinical parameters. As selenoprotein expression is a modifiable parameter mainly dependent on selenium intake, the personalized correction of a diagnosed deficiency should be investigated in future studies to improve CRC patient survival.

## 1 Introduction

Colorectal cancer (CRC) is the third leading cancer-related cause of death worldwide and represents a major public health issue [1]. CRC is a complex disease with several established modifiable and unmodifiable risk factors such as age, male sex, family history of CRC, genetic factors, inflammatory bowel disease, diabetes, obesity, smoking, physical inactivity, excessive alcohol consumption, low calcium intake and high consumption of red meat [[2], [3], [4], [5]]. Most CRCs are sporadic and usually develop in a slow progression from normal epithelium to precancerous low-risk and high-risk adenomas to invasive cancer [6]. This offers significant opportunities for preventive screening, early intervention by removal of precancerous adenomas and supportive adjuvant therapy [7,8]. Early detection of CRC is an important issue since stage at diagnosis remains the most important prognostic factor [9,10]. As CRC is one of the most preventable cancers, population-wide screening programs are recommended as they offer the potential to detect early lesions and perform endoscopic removal of adenomas, thereby contributing to the reduction of CRC incidence and mortality [[11], [12], [13]].

The ongoing “Colorectal Cancer Study of Austria” (CORSA) is one such study, and part of a large prospective population-wide program in the Austrian federal state of Burgenland, aiming to identify survival-relevant factors in CRC [14]. Among the promising candidates is the trace element selenium (Se), as it affects risk and course of intestinal diseases [15,16]. As the Se content and its bioavailability in the soils used for agricultural food production differ strongly around the world, many populations are at risk of Se deficiency, including Europe and large parts of Asia and Africa [17,18]. A sufficiently high intake is essentially needed for the biosynthesis of selenoproteins that are characterized by containing the 21st proteinogenic amino acid selenocysteine. In humans, they are encoded by 25 separate genes [19]. Certain members of the selenoprotein family are involved in antioxidative activities, thyroid hormone metabolism, protein quality control and other central pathways of relevance for cancer prevention and promotion [20]. Prospective epidemiological studies have indicated that low Se concentrations in blood or deficiency in the circulating Se transporter selenoprotein P (SELENOP) predispose to an increased CRC risk [21,22], and shorter survival [23].

However, it is also known that tumors tend to accumulate the trace element, as tumor cells require abundant expression of selenoproteins for their proliferation and survival, especially to protect against oxidative damage and to resist ferroptosis [24,25]. Recently, this property has been shown to contribute to the development of metastases and the survival of disseminated tumor cells [26]. Therefore, it is an unsolved question whether Se deficiency or replete Se status at the time of diagnosis is beneficial for an acutely diseased CRC patient, as the limiting trace element and several selenoenzymes support the functions and activity of the patient's immune, endocrine and central nervous system as well as the survival, proliferation and metabolism of tumor cells [16,27,28].

In view of the essentiality of Se and the central biochemical and physiological roles of selenoproteins, we hypothesized that a depressed Se status and low expression of selenoproteins at time of CRC diagnosis may negatively affect survival prospects. Moreover, we speculated that a combination of three markers of Se status, namely total-Se, activity of the extracellular selenoprotein glutathione peroxidase-3 (GPx3) and concentration of SELENOP provides more accurate prognostic information than a single marker alone, as shown before in breast cancer [29]. In order to test these hypotheses, we quantified all three Se status biomarkers in participants of CORSA, including control subjects and patients with adenomas or CRC, integrated the data with a large data base of healthy European adults, and analyzed Se status in relation to mortality over a time course of up to 15 years of follow-up.

## 2 Material & methods

### 2.1 Study details

CORSA is an ongoing multicenter study since 2003, recruiting participants in cooperation with the province-wide CRC screening program “Burgenland Prevention Trial of Colorectal Disease with Immunological Testing” (B-PREDICT) using a fecal immunochemical test (FIT) as initial screening tool [14]. FIT-positive participants of B-PREDICT received a complete colonoscopy and were invited to take part in CORSA. Additional participants were recruited at four hospitals in Vienna. CORSA includes men and women aged between 30 and 90, and excludes patients diagnosed with hereditary CRC syndromes or with any previous cancer history. Written informed consent was obtained from all study participants, and EDTA plasma and a stool sample were collected in a biobank. Information on demographic (e.g. age at diagnosis, weight, height) and lifestyle factors (including diabetes status, alcohol consumption, and smoking status) was obtained through self-assessment using the basic CORSA questionnaire, as previously described [14]. All CRC patients were diagnosed as histologically confirmed stage I-IV sporadic CRC. Patients diagnosed with adenomatous tubular polyps *≥* 1 cm, adenomatous tubulo-villous polyps and/or adenomatous villous polyps were categorized into the adenomas group. Blood samples were obtained from participants prior to surgery or any radio- or chemotherapy, processed within 4 h following standardized protocols, and stored as plasma at −80 °C until further processing.

### 2.2 Assessment of clinical data and follow up

Clinical data were abstracted from medical records and processed in a structured database following standardized documentation guidelines and according to the EU General Data Protection Regulation (GDPR). The CORSA databank comprises structured information on diagnosis, treatment, histology, progression (recurrence and metastasis), and survival data [14]. Follow-up on clinical data is regularly performed. Survival data, including information on confirmed date of death, date of last contact, and information on missing or incorrect data input, are provided through a biennial clinical data abstraction from the IT database of the Medical University of Vienna, “Allgemeines Krankenhaus Information Management” (AKIM), in cooperation with Statistics Austria. In addition, CORSA survival data are abstracted in cooperation with the Main Association of Austrian Social Insurance Institutions (“Hauptverband der österreichischen Sozialversicherungsträger”). Hereby, social insurance number, first and last name, date of birth, and sex were received for all participants, and were processed through a database pipeline.

### 2.3 Assessment of selenium status biomarkers

#### 2.3.1 Total plasma selenium

Total-Se was analyzed using total reflection X-ray fluorescence (TXRF) analysis using a TXRF device (S4 T-STAR, Bruker nano GmbH, Berlin, Germany), as described [29]. In brief plasma was diluted 1:2 with a gallium standard (1000 μg/L), 8 μL of the mixture was applied on a polished quartz glass slide (Bruker nano) and dried at 37 °C overnight. The TXRF analysis was performed the next day. A Seronorm standard (Sero AS, Billingstad, Norway) served as control. The inter- and intra-assay coefficients of variation (CV) were below 10 % during the measurements.

#### 2.3.2 Selenoprotein P

Selenoprotein P concentrations were determined by a chemiluminescent immunoassay (CLIA) using an automated system (iSYS automat, Immunodiagnostic Systems Holdings Ltd; ids), with monoclonal antibodies against SELENOP as described [30]. Three samples spanning the working range of the assay were obtained (selenOmed GmbH, Berlin, Germany) and included in each analytical run as controls. The inter- and intraassay CV were below 5 % during the analyses.

#### 2.3.3 Glutathione peroxidase 3

The enzymatic activity of GPx3 was determined by a coupled enzyme reaction, by following the decrease of NADPH, as described earlier [31]. In brief, NADPH consumption by added glutathione reductase is reflected in a reduction of UV absorption at 340 nm, which in turn is proportional to the reduction of oxidized glutathione from the GPx3 catalyzed reaction, and thus to its enzymatic activity. GPx3 activity is expressed as unit (U), corresponding to the amount of enzyme that catalyzes the oxidation of 1.0 nmol of NADPH per min at 25 °C. The samples were measured in triplicates, and controls were analyzed in parallel, yielding inter- and intraassay CV of below 15 %.

#### 2.3.4 SELENOP autoantibodies

Autoantibodies to SELENOP (SELENOP-aAb) in the plasma samples were detected and assessed as previously described [32]. In brief, 5 μL plasma was incubated with a fusion protein consisting of a secreted reporter enzyme fused in frame to a recombinant SELENOP variant (selenOmed GmbH). Samples were incubated over night at 4 °C, and the formed immune complexes (SELENOP-aAb bound to the fusion protein) were precipitated with protein A sepharose, washed and analyzed for reporter activity in a luminometer. Luminescence corresponding to the SELENOP-aAb concentration was recorded as relative lights units (RLU) and analyzed in relation to background signals. Inter- and intraassay CV using a positive sample as standard were below 20 % during the analyses.

### 2.4 Statistics

#### 2.4.1 Comparisons and correlations

Wilcoxon rank sum test was used to test differences in continuous variables, Fischer's exact test was used to test differences between categorical variables in a 2 x 2 contingency format, and Pearson's Chi-squared test was used to test differences in categorical variables with more categories. Correlation between the Se markers was tested with Spearman's rank correlation and visualized by linear regression plots with 95 % confidence intervals (CI).

#### 2.4.2 Classification of selenium marker status

Se markers were determined and subsequently categorized into two groups (low versus high). The overall study median for each marker was calculated as threshold, with patients below the median assigned to the low group and patients at or above the median assigned to the high group.

#### 2.4.3 Subgroup clustering of patients

A k-means clustering analysis was performed to identify subgroups within the dataset based on Se status. The clustering variables included Se, SELENOP, and GPx3. Prior to clustering, the data were standardized using z-score normalization. The optimal number of clusters was determined using the elbow method, based on the within-cluster sum of squares. K-means clustering was then applied with three clusters (k = 3), using 25 random starts to ensure stability of the solution. To visualize the clustering results, principal component analysis (PCA) was conducted, and the first two principal components were plotted, color-coded by cluster assignment. Additionally, cluster-specific distributions of Se, SELENOP, and GPx3 were examined using boxplots.

#### 2.4.4 Survival probability

Survival probability was visualized with Kaplan-Meier plots. The log-rank test was used to detect differences between the groups, and between the Se marker status group, i.e. low Se vs. high Se or low SELENOP vs. high SELENOP. Hazard ratios (HR) along with 95 % Confidence Interval (CI) were calculated using Cox regression models, crude and multivariable adjusted for potential confounders of mortality. The first model included the respective marker of Se status only, the second model was adjusted for age, sex, BMI, smoking, alcohol consumption and tumor localization (in the CRC subset). HR and 95 % CI of the associations were visualized, and restricted cubic splines with knots at the 10th, 50th and 90th centiles were implemented to allow nonlinearity. The linearity of the associations was investigated by comparing the restricted cubic spline models to nested linear models using the likelihood ratio test, whereby P < 0.05 was considered a nonlinear association.

#### 2.4.5 Classification of autoimmunity to SELENOP

Patients were assigned as SELENOP-aAb positive or negative based on the obtained signals from the plasma sample as described above. Classification was carried out after finishing all measurements by applying a mathematical outlier criterion. Based on the assumption that SELENOP-aAb are prevalent in less than 50 % of patients, the arithmetic means of the lowest 50 % of signals per measured 96-well plate was calculated and defined as background with an assigned baseline binding index (BI) of BI = 1.0. All values equal or above 5-fold of this signal, i.e., BI ≥ 5 were considered as SELENOP-aAb positive. Distribution of the resulting BI of all results was displayed by dot-plots and prevalence was calculated and compared.

#### 2.4.6 Software

All statistical analyses were conducted using R (Version: 4.5.0) on the RStudio environment. The following packages were used for data structure; Dplyr (ver. 1.1.4), tidyr (ver. 1.3.1), tidyverse (ver. 2.0.0), and visualization ggplot2. Table generation was performed with gtsummary (ver. 2.0.4) or Hmisc (ver. 5.2–2), and rms (ver. 7.0–0) was used for visualization of restricted cubic spline regression models. The package survival (ver. 3.8–3) was used for survival analysis, and survminer (ver. 0.5.0) for visualization of results. Kmeans clustering and principal components analysis was conducted with stats (ver 4.4.1). In all the analyses P < 0.05 was considered as significant.

### 2.5 Ethical aspects

Written informed consent was obtained from all study participants prior to analyses. Compliance with the 1964 Declaration of Helsinki, the Austrian Drug Law (Arzneimittelgesetz, AMG), and the requirements of Good Clinical Practice of the European Community (CPMP/ICH/135/95) were ensured. The CORSA study was approved by the institutional review boards in Vienna (EK 33/2010 and EK 1160/2016).

## 3 Results

A total of 519 participants were included in the final analyses, consisting of 153 controls, 255 patients with adenomas, and 111 with CRC. The median age was 65 years, median BMI was 27.8, and 63 % of the participants were males. The median follow-up was 5424 days (almost 15 years), with a total of 210 deaths (40 %). The median Se, SELENOP and GPx3 levels were 65.7 μg/L, 2.7 mg/L and 201.2 U/L, respectively (Table 1). Collectively, the anthropometric characteristics and other risk factors were comparable and similarly distributed across the three groups.

**Table 1:** Study characteristics of the CORSA-derived colorectal neoplasia cohort.

Characteristic	Overall n = 519^a^	Controls n = 153^a^	Adenoma n = 255^a^	CRC n = 111^a^
**Patient age [years]**	65.0 (16.0)	64.0 (19.0)	65.0 (15.0)	67.0 (16.0)
**Sex**				
Male	327 (63 %)	90 (59 %)	164 (64 %)	73 (66 %)
Female	192 (37 %)	63 (41 %)	91 (36 %)	38 (34 %)
**BMI**	27.8 (5.7)	27.3 (4.7)	28.3 (5.6)	27.8 (6.1)
**Smoking**				
Smoker	67 (13 %)	14 (9.2 %)	34 (13 %)	19 (17 %)
Former smoker	178 (34 %)	57 (37 %)	84 (33 %)	37 (33 %)
Never smoker	274 (53 %)	82 (54 %)	137 (54 %)	55 (50 %)
**Selenium [μg/L]**	65.7 (17.6)	66.1 (19.6)	66.1 (16.1)	64.0 (19.1)
**SELENOP [mg/L]**	2.7 (1.0)	2.9 (0.9)	2.8 (1.0)	2.4 (0.9)
**GPx3 activity [U/L]**	201.2 (53.8)	207.3 (62.9)	198.4 (47.4)	203.8 (60.7)
**Alcohol**				
No alcohol consumption	159 (31 %)	47 (31 %)	75 (29 %)	37 (33 %)
Former consumption	43 (8.3 %)	14 (9.2 %)	18 (7.1 %)	11 (9.9 %)
Consumption of alcohol	317 (61 %)	92 (60 %)	162 (64 %)	63 (57 %)
**Follow-up days**	5424 (2398)	5564 (1662)	5512 (1963)	4738 (3756)
**Death**	210 (40 %)	48 (31 %)	100 (39 %)	62 (56 %)

a Median (IQR) or n(%), CRC; colorectal cancer, BMI; body mass index, SELENOP; selenoprotein P, GPx3; glutathione peroxidase 3.

**Fig. 1:**
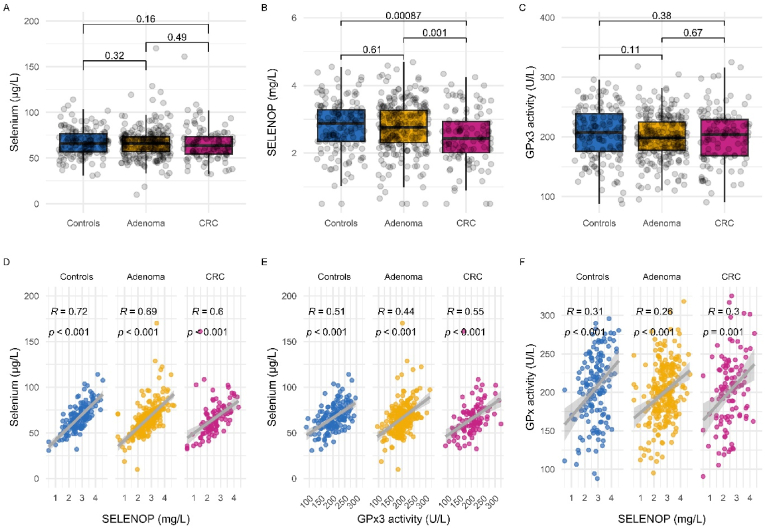
Comparison and correlation of Se markers. **A)** Comparison of total Se in the different groups, Wilcoxon rank sum test (one data point with Se > 300 μg/L in CRC was removed to improve the scaling of the figure). **B)** Comparison of SELENOP concentrations in the different groups, Wilcoxon rank sum test. **C)** Comparison of GPx3 activity in the different groups, Wilcoxon rank sum test. **D) - F)** Correlation of the three different Se markers in the different groups. Spearman's R is reported, while the grey line with 95 % confidence intervals is derived from linear regression analysis. CRC; colorectal cancer, Se; selenium, SELENOP; selenoprotein P, GPx3; glutathione peroxidase 3.

The groups did not differ in total-Se or GPx3 activity (Fig. 1). However, the patients with CRC had significantly lower SELENOP levels compared to adenoma patients or controls (Fig. 1B). All three Se markers showed significant positive pairwise correlations in all subject groups, supporting the notion of a general Se-deficiency (Fig. 1D–F).

**Fig. 2:**
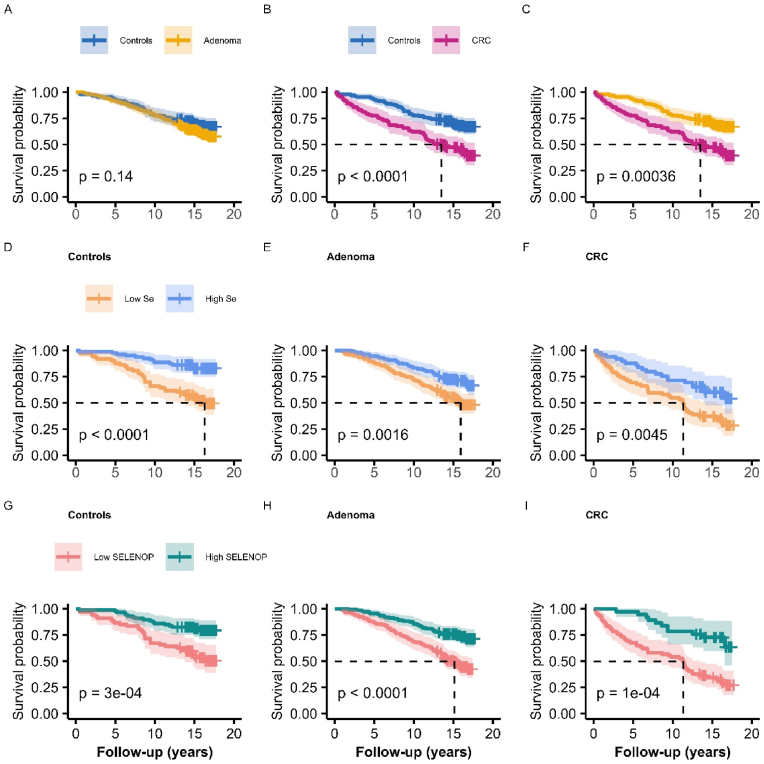
Kaplan-Meier plots for overall survival for analyses A–I: **A)** Subjects with adenomas compared to controls. **B)** Patients with colorectal cancer (CRC) compared to controls. **C)** Patients with adenoma versus CRC. **D)** Controls stratified by median Se status. **E)** Patients with adenomas stratified by median Se status. **F)** Patients with CRC stratified by median Se status. **G)** Controls stratified by median SELENOP status. **H)** Patients with adenomas stratified by median SELENOP status. **I)** Patients with CRC stratified by median SELENOP status. Median plasma Se; 65.7 μg/L, and median SELENOP concentration; 2.7 mg/L. Se; selenium, SELENOP; selenoprotein P.

Survival probability was compared between the groups (Fig. 2). There was no significant difference in overall survival between the adenoma and control groups (Fig. 2A). Overall survival was lower in the CRC group compared to the control and adenoma groups (Fig. 2B and C). All patients were further stratified into low Se (Se < 65.7 μg/L) versus high Se (Se ≥ 65.7 μg/L). Subjects within the low Se classification had significant lower overall survival in all groups (Fig. 2D–F). Similarly, when the subjects were stratified into low SELENOP (SELENOP <2.7 mg/L) versus high SELENOP (SELENOP ≥2.7 mg/L), the low SELENOP strata had a lower overall survival in all groups (Fig. 2G–I).

Associations of concentrations of Se, SELENOP and activity of GPx3 with all-cause mortality were analyzed in all three patient groups, and estimated HRs and 95 % CI for death from Cox proportional hazards models are provided (Table 2). To allow for better comparability of effect sizes across biomarkers with different units and distributions, all variables were z-score standardized prior to analysis.

**Table 2:** Association of selenium markers with mortality.

	Univariate			Fully adjusted^b^		
Characteristic	N	HR^a^	95 % CI^a^	N	HR^a^	95 % CI^a^
*Control*						

**Selenium** (per SD increase)	153	0.47	0.35, 0.65	153	0.64	0.45, 0.90

**SELENOP** (per SD increase)	153	0.51	0.39, 0.67	153	0.62	0.46, 0.83
**GPx3 activity** (per SD increase)	153	0.67	0.51, 0.88	153	0.84	0.60, 1.16

*Adenoma*						

**Selenium** (per SD increase)	255	0.70	0.55, 0.91	255	0.88	0.69, 1.12
**SELENOP** (per SD increase)	255	0.63	0.52, 0.77	255	0.73	0.59, 0.90
**GPx3 activity** (per SD increase)	255	0.83	0.68, 1.01	255	0.93	0.75, 1.16

*CRC*						

**Selenium** (per SD increase)	111	0.79	0.49, 1.28	111	1.05	0.68, 1.62
**SELENOP** (per SD increase)	111	0.54	0.42, 0.71	111	0.64	0.49, 0.84
**GPx3 activity** (per SD increase)	111	0.68	0.51, 0.89	111	0.74	0.55, 1.00

a HR; hazard ratio, CI; confidence interval, SD; standard deviation, CRC; colorectal cancer, Se; selenium, SELENOP; selenoprotein P, GPx3; glutathione peroxidase 3.

b Adjustment for age, sex, BMI, smoking status, alcohol consumption, and tumor localization (in the CRC group).

**Fig. 3:**
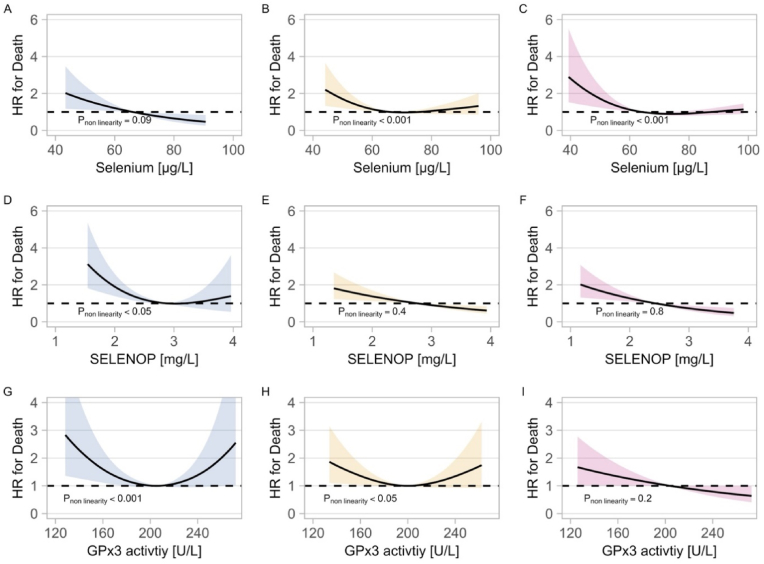
Associations of selenium (Se), SELENOP and GPx3 activity with survival. Linear regression models with three knots at centiles 10, 50 and 90 were calculated to account for non-linearity. All models are fully adjusted. Blue represents the control group, yellow the adenoma group, and red the colorectal cancer (CRC) group. **A) - C)** The associations of Se with death in the different groups. **D) - F)** The associations of SELENOP with death in the different groups. **G) - I)** The associations of GPx3 with death in the different groups. HR; hazard ratio, SELENOP; selenoprotein P, GPx3; glutathione peroxidase 3.

The visual associations as univariate models, using restricted cubic spline regression with three knots in the case of a non-linear association, and linear models in the case of a linear association, are provided as supplement (Supplementary Figure 1). The results did not notably differ in the fully adjusted models (Fig. 3). The control and adenoma models were adjusted for age, sex, BMI, smoking status, alcohol consumption, and the CRC model was further adjusted for cancer localization. All Se markers showed an inverse association with death in all groups. For SELENOP, there was a consistent inverse linear association in the adenoma and CRC groups, with a higher SELENOP concentration being associated with a lower HR for death.

Next, the associations of Se, SELENOP, and GPx3 with death were tested in a combined analysis as a univariate model, fully adjusted by age, sex, BMI, smoking status, alcohol consumption and patient groups. Selenium and GPx3 showed a non-linear J-shaped inverse association with death, while SELENOP showed an inverse linear association with death over the complete concentration range (Supplementary Table 1, Supplementary Figure 2).

**Fig. 4:**
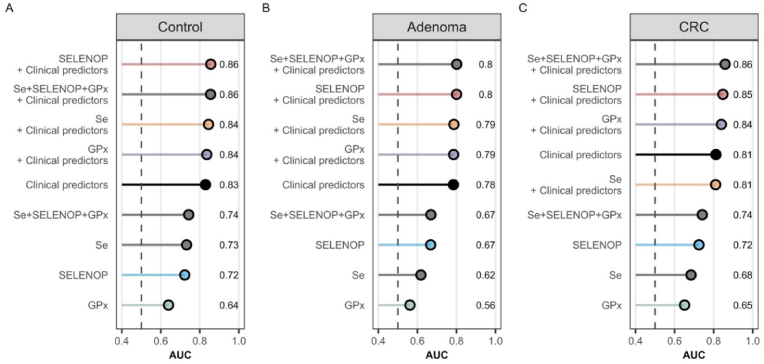
Predictive values of different models based on Se or SELENOP concentrations and GPx3 activity. **A)** Ranked area under the curve (AUC) for the control subject model. **B)** Ranked AUC for the adenoma patient model. **C)** Ranked AUC for the colorectal cancer (CRC) patient model. An AUC of 0.5 represents a random predictor, whereas an AUC of 1.0 represents a prediction model with 100 % specificity and 100 % sensitivity. Covariables: age, sex, BMI, smoking status, alcohol consumption, and tumor localization (in the CRC group).

Predictive models were calculated for the individual Se parameter clinical predictors and comminated parameter (Fig. 4). The combined models with triple Se parameter and clinical predictor showed the highest areas under the curve (AUC: 0.86, 0.8, 0.85) in all three groups compared to the clinical predictor only models (AUC: 0.83, 0.78, 0.81). The combined models with SELENOP as a single Se marker showed higher AUC in all three groups compared to the clinical predictor only models (AUC: 0.86, 0.8, 0.85 vs. 0.83, 0.78, 0.81). The combination of the triple Se parameter did not show an increased AUC in the control and adenoma patient groups compared to the single parameter SELNEOP. In CRC only, the triple Se model had a 0.1 higher AUC compared to SELENOP adjusted for covariates.

**Fig. 5:**
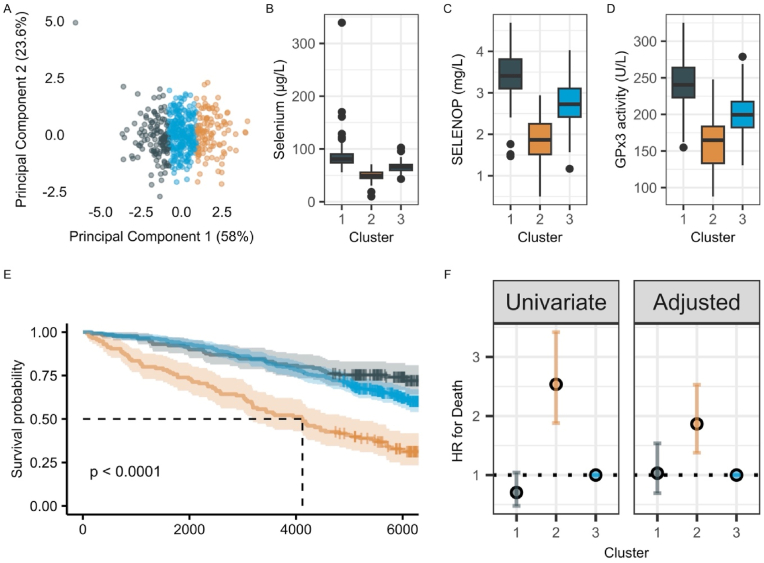
Cluster analysis of Se markers. **A)** Shows the principal component analysis after standardization of the Se marker values. The values are grouped into three clusters. **B) -D)** Shows the distribution of the Se markers for the different clusters. **E)** Displays the Kaplan-Meier curve showing the overall survival of the clusters**. F)** Displays the hazard ratio (HR) for death for clusters 1 and 2 in comparison to cluster 3.

Using k-means clustering analysis the three markers Se, SELENOP, and GPx3 were clustered and visualized by principal component analysis (PCA); 81.6 % of the variance might be captured by PC1 and PC2 (Fig. 5A, Supplementary Table 2). The resulting cluster 1 included the highest Se, SELENOP and GPx3 levels (median, Se: 81 μg/L, SELENOP: 3.4 mg/L, GPx3: 240 U/L), cluster 2 showed the lowest values (median, Se: 49 μg/L, SELENOP: 1.85 mg/L, GPx3: 165 U/L), and cluster 3 resided between clusters 1 and 2. The overall survival was significantly lower in cluster 2 as compared to cluster 1 or cluster 3 (Fig. 5).

**Fig. 6:**
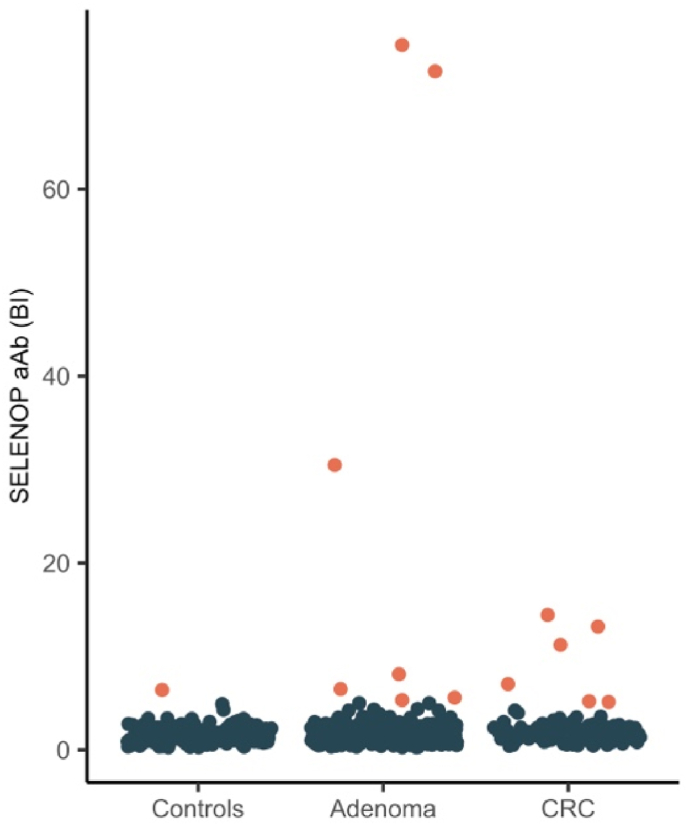
Analysis of autoimmunity to the Se transporter SELENOP (SELENOP autoantibodies; SELENOP-aAb). The prevalence of SELENOP-aAb differs between the groups. Black: Subjects negative for SELENOP-aAb. Orange: Subjects positive for SELENOP-aAb. BI, binding index; CRC, colorectal cancer.

Patients were also analyzed for SELENOP-aAb, to identify whether tumorigenesis associates with a higher prevalence of autoimmunity to SELENOP, as observed before in breast cancer [33]. The prevalence of SELENOP-aAb differed between the groups and was 0.7 % in controls, and 5.4–5.5 % in patients with CRC or adenomas (Fig. 6). The total number of SELENOP-aAb positive subjects was relatively small, precluding further statistical analyses for their relevance regarding survival (Supplementary Table 3).

## 4 Discussion

Low Se status has been associated with increased cancer risk in previous analyses, supporting the notion that Se deficiency constitutes a risk factor for CRC development [16,23,34]. However, as tumor cells take advantage of selenoproteins to protect from oxidative damage and death by ferroptosis, a better Se status may support tumorigenesis and metastasis once a cancer is developing and the diagnosis is made [16,26,34]. In this study, we present data from a large observational prospective study on the importance of Se status at the time of CRC diagnosis for survival. The study was conducted in a European population with relatively strong Se deficiency, reflected in the data by the linear associations between plasma Se and selenoprotein concentrations across the full concentration ranges. The three study groups were recruited from a defined geographic area in response to the same screening result, i.e., a positive FIT, and displayed highly similar anthropometric characteristics and CRC risk factors. Based on coloscopy, the participants were grouped into healthy controls, patients with adenomas or CRC. The longitudinal comparison yielded the expected shorter life expectancy for CRC as compared to adenomas or controls [35], and highlighted the importance of Se status for survival in all groups, i.e., in the controls and in both groups of patients.

This insight was enabled by the relatively long follow-up period of almost 15 years, and the senior age of most of the participants at enrollment causing a high mortality event rate. Among the three Se status markers analyzed, SELENOP deficiency emerged as the most stringently associated parameter for mortality. Notably, the effect size was consistently high across all groups, and the prognostic value of taking the Se status into consideration was similar when using SELENOP alone, or a combination of all three Se biomarkers. The results accord with a recent large-scale prospective observational study of German adults, indicating higher mortality from all major diseases in subjects with SELENOP deficiency [36]. Our combined analysis using all three Se markers indicated that prognosis accuracy can be improved via a clustered approach implementing the full set of data, as for a similar previous analysis in breast cancer [29]. Finally, the analysis of SELENOP-aAb highlighted that tumorigenesis may have induced autoimmunity to SELENOP, as observed before in breast cancer [33], or as observed in response to severe inflammation following burn injury [37]; however, the few SELENOP-aAb positive subjects in this study precluded further analyses of their relevance. Collectively, the data highlight a positive association of Se status with survival, both in controls and CRC patients, in particular regarding SELENOP concentrations.

The data are in accordance with several previous reports analyzing total serum or plasma Se concentration in observational cohort studies, collectively indicating increased cancer risks and cancer mortality associated with the lowest categories of Se exposure [38]. Among the different organs, the intestinal tract appears particularly sensitive to Se, probably because of the protection elicited by selenoproteins on epithelial damage, gut barrier integrity and excessive oxidative stress, as evidenced in studies on CRC-predisposing inflammatory bowel diseases [16,39].

Accordingly, the large European Prospective Investigation into Cancer and Nutrition (EPIC) cohort reported an increased risk for CRC in subjects with low SELENOP or Se concentrations [21,22]. A follow up analysis indicated an inverse trend of prediagnostic Se and SELENOP status with overall and CRC-specific mortality, albeit without reaching statistical significance [23]. Two main reasons have likely contributed to the stronger and highly significant associations detected in the present study. While the average age in this study and EPIC was similar (65.0 and 62.4 years, respectively), EPIC analyzed a prediagnostic sample, i.e., the Se status was determined from one sample taken on average 46 months before CRC diagnosis, whereas the current study assessed Se status at time of diagnosis. In addition, the follow up time period was longer in the current analysis than in EPIC (178 versus 113 months), and Se status was considerably lower in the current analysis than in EPIC (EPIC [23], 3rd quintile: mean (SD) for Se; 82.6 (2.8) μg/L, and SELENOP; 4.3 (0.7) mg/L, versus CORSA; median (IQR) for Se; 65.7 (17.6) μg/L, and SELENOP; 2.7 (1.0) mg/L). Among all the studies analyzed so far in our laboratory, the participants of CORSA were exceptionally low in these Se status biomarkers, given that the subjects had not suffered an acute injury or severe infection at the time of sampling. The high fraction of CORSA participants with considerable Se deficiency (>50 % below 70 μg/L) is also reflected in the strong linear correlations of Se with GPx3 and SELENOP concentrations. No such correlation of total-Se with selenoproteins is usually observed in countries with high baseline Se intake, in particular in the USA, which provide the majority of data for large-scale meta-analyses on the potential role of Se in cancer [38,40]. A saturated expression of the extracellular selenoproteins is widely used to define Se sufficiency on a population level, as suggested by different nutritional societies in Europe [17,41]. Once saturation is achieved, there is no plausible reason for Se supplementation, and health aspects related to Se deficiency like cancer or cardiovascular disease risks become independent from status [40].

In view of the clear-cut results in this study, two aspects become particularly relevant and important, namely the potential molecular mechanism behind the findings, and the potential requirement for Se supplementation to correct an identified Se deficiency and thereby to attempt reducing CRC mortality and associated disease risks.

The literature on the potential molecular roles of Se in the different stages of carcinogenesis, i.e., malignant transformation, tumor initiation, development, growth, ferroptosis resistance, and metastasis, is very large, and a number of these processes and pathways have been identified to be strongly affected by selenoprotein deficiency [15,42,43]. Prime candidates for a direct role in redox homeostasis in CRC are the selenoproteins of the GPX and thioredoxin reductase (TXNRD) families, along with SELENOH and SELENOW [15,42,44,45]. Besides redox control, biosynthesis and quality control of secretory and membrane-associated proteins depend on the function of a set of at least seven cancer-relevant ER-resident selenoproteins, namely SELENOF, I, K, M, N, S, and T [46,47]. Cancer metastasis strongly relies on survival of the migrating cells, constantly at risk of detection by the immune system, circulating under high energy requirements, and being exposed to micro-environmental and intracellular inducers of ferroptosis [48,49]. Hence, expression of GPx4 and control of mitochondrial function, e.g. by SELENOO, are central Se-dependent components for successful detachment, cell migration and establishment of metastases [26,50,51].

Besides these biochemical pathways, recent studies have also highlighted the impact of Se and certain selenoproteins on the endocrine control of hormone-responsive tumors, i.e., prostate and gynecological malignancies, and in particular breast and thyroid cancer [34,[52], [53], [54]]. Collectively, a complex picture on diverse interactions emerges, with intracellular signaling, proliferation, redox control, endocrine signaling, extracellular matrix interaction, and migration as well as the interaction with the immune system and new microenvironment all being affected by the activity and expression levels of selenoproteins, albeit at different stages and to variable degrees [26,28,42,44,55,56].

Accordingly, Se deficiency forces the host system to prioritize certain selenoproteins during biosynthesis over other selenoproteins, i.e. to apply a triage strategy, and not all of the relevant members will be expressed to their full activity, with apparent adverse effects on survival odds [57]. In the case of intestinal tumors, additional impact of Se on the microbiota, and active competition between the host and the microbes for the limiting trace element may further aggravate Se deficiency, with assumed relevance for CRC development [58,59]. An insufficient Se supply may shift the microbial flora out of balance, inducing dysbiosis, in particular when pro- and eukaryotic cells depend on the same decreasing Se pool [60].

Preclinical model systems have indicated that the molecular role of SELENOP in CRC appears to be particularly complex, stage- and cell type-specific [16]. The majority of circulating SELENOP is of hepatic origin, but expression of SELENOP is widespread in the organism, including the central nervous, endocrine and gastrointestinal system [61]. Mouse and cell culture studies indicated an important role of intestinal SELENOP expression for the progression of adenomas to carcinoma, e.g. by modulating WNT signaling through LDL receptor-related proteins, i.e., members of the same family as the established SELENOP-specific uptake receptors [28,62]. The transgenic models identified the prime contribution of intestinal SELENOP expression to inflammation in the local microenvironment, affecting genomic stability, and epithelial stem cell characteristics [55]. In biopsies of colitis patients, SELENOP levels were low and associated inversely with disease severity [56]. The underlying mechanism for the progressively suppressed SELENOP levels, both systemically and locally in the intestine, likely involves a down regulation of its transcripts and of other rate limiting components of the general selenoprotein biosynthesis machinery in response to inflammation [63,64]. Alternatively, an accumulation of Se in cancer tissue has been described, but it is unlikely that the preferential uptake from the circulation can be made responsible for the declining SELENOP status in blood of CRC patients [65].

When it comes to chemoprevention and Se supplementation, the present data derive mainly from studies that have been conducted in the USA, where Se deficiency is rare [66]. In agreement with mainly negative results, it has been widely consented in recent years that supplemental intake is of no health benefit when no Se deficiency is present [38,40,66]. Supplemental Se on top of a sufficient baseline intake seems to even come with a small but relevant risk of supporting type 2 diabetes mellitus development [67], and has been reported to be associated with an increased risk for high grade prostate cancer [68]. Yet again, these results were from studies with well-supplied subjects, which is partly in direct disagreement with observational studies analyzing the same matrix and cancer type in the EU [69].

Unfortunately, no large prospective cancer prevention studies with supplemental Se intake have been conducted in areas with as low a baseline Se status as in the current study, and even though it is likely that correcting a strong Se deficit would be of health benefit, the final experimental proof from a sufficiently long and powered active intervention trial is missing. Given the health risks for many major diseases that apparently increase with insufficient SELENOP expression [36], the advantages will most likely outbalance the potential risks, especially when the degree of Se deficiency is as strong as in this study. A general feasibility of increasing Se supply safely by a country-wide measure has been shown by Finland, where all fertilizers become systematically enriched, yielding improved Se concentrations in agricultural products and higher intake by the Finish population [70]. The health potential of correcting a Se deficit and improving SELENOP concentrations has been reported from a Scandinavian randomized controlled trial, where elderly subjects received supplemental Se and coenzyme Q10 or placebo, and displayed lower cardiovascular and total mortality risks and better survival on follow-up [71,72]. Tumorigenesis or cancer-related death was unfortunately not in the focus of this intervention trial.

Among the strengths of this study is the recruitment strategy enrolling FIT-positive subjects within a CRC screening program, followed by colonoscopy to diagnose and stratify three disease groups with similar anthropometrics. Moreover, the follow-up period was relatively long. The exposure Se status was assessed in a comprehensive manner with three markers, indicating particularly low status values even within Europe. Among the limitations are the pure observational nature of the study, precluding causal inferences, lack of knowledge on treatment modalities and their potential impact on Se status and mortality, and the analysis of a single plasma sample only. Moreover, our analysis was focused on biomarkers of Se status, without considering other micronutrients and their potential interactions. Yet, by assessing three complementary markers with varying half-lives and representing different aspects of Se physiology, the risk of misclassification of Se status is low.

We conclude that these data indicating elevated mortality risk with low SELENOP status at the time of cancer diagnosis further emphasize the need to avoid Se deficiency and insufficient Se intake. The strongest factor for an inappropriate expression of selenoproteins is given by low Se supply, i.e., selenoprotein deficiency is an unfavorable setting that needs to be diagnosed, actively corrected and monitored to reduce disease and mortality risks from an easily modifiable and addressable nutritional insufficiency. Yet, supplemental intake in pharmacological concentrations exceeding the UL for Se are not encouraged and such recommendations cannot be based on the current study and its results.

## CRediT authorship contribution statement

**Stefanie Brezina:** Writing – original draft, Methodology, Investigation, Formal analysis, Data curation, Conceptualization. **Thilo Samson Chillon:** Writing – original draft, Visualization, Software, Methodology, Investigation, Formal analysis, Data curation, Conceptualization. **Sabrina Asaad:** Writing – review & editing, Methodology, Investigation, Formal analysis, Data curation. **Andreas Maieron:** Writing – review & editing, Validation, Resources, Formal analysis. **Julian Prosenz:** Writing – review & editing, Validation, Resources, Methodology, Investigation. **Julian Seelig:** Writing – review & editing, Validation, Methodology, Formal analysis. **Kamil Demircan:** Writing – review & editing, Validation, Software, Methodology, Formal analysis, Data curation. **David J. Hughes:** Writing – review & editing, Validation, Formal analysis, Data curation. **Andrea Gsur:** Writing – original draft, Supervision, Project administration, Investigation, Funding acquisition, Conceptualization. **Lutz Schomburg:** Writing – original draft, Supervision, Resources, Project administration, Methodology, Investigation, Funding acquisition, Conceptualization.

## Ethical approval and consent to participate

Written consent was obtained from all study participants and all studies were approved by the corresponding Institutional Review Board. Compliance with the 1964 Declaration of Helsinki, the Austrian Drug Law (Arzneimittelgesetz, AMG) and the requirements of Good Clinical Practice of the European Community (CPMP/ICH/135/95) were ensured. The CORSA study was approved by the institutional review boards (EK 33/2010 and EK 1160/2016).

## Funding

This study was funded by the “Österreichische Forschungsförderungsgesellschaft” FFG BRIDGE (grant 880626, to Andrea Gsur) and was supported by COST Action CA17118. Research in the laboratory of L.S. is supported by Deutsche Forschungsgemeinschaft (DFG), LocoTact CRC/TR296.

## Declaration of competing interest

The authors declare the following financial interests/personal relationships which may be considered as potential competing interests: Lutz Schomburg reports financial support was provided by Charite - Universitätsmedizin Berlin. Lutz Schomburg reports financial support was provided by Deutsche Forschungsgemeinschaft DFG. Andrea Gsur reports financial support was provided by Austrian Academy of Sciences. Lutz Schomburg reports a relationship with selenOmed GmbH that includes: equity or stocks. If there are other authors, they declare that they have no known competing financial interests or personal relationships that could have appeared to influence the work reported in this paper.

## Data Availability

Data will be made available on request.

## References

[1] Sung H., Ferlay J., Siegel R.L., Laversanne M., Soerjomataram I., Jemal A., Bray F. (2021). Global cancer Statistics 2020: GLOBOCAN estimates of incidence and mortality worldwide for 36 cancers in 185 countries. CA Cancer J. Clin..

[2] Papier K., Bradbury K.E., Balkwill A., Barnes I., Smith-Byrne K., Gunter M.J., Berndt S.I., Le Marchand L., Wu A.H., Peters U. (2025). Diet-wide analyses for risk of colorectal cancer: prospective study of 12,251 incident cases among 542,778 women in the UK. Nat. Commun..

[3] Postwala H., Shah Y., Parekh P.S., Chorawala M.R. (2023). Unveiling the genetic and epigenetic landscape of colorectal cancer: new insights into pathogenic pathways. Med. Oncol..

[4] Molla M.D., Symonds E.L., Winter J.M., Debie A., Wassie M.M. (2024). Metabolic risk factors of colorectal cancer: umbrella review. Crit. Rev. Oncol. Hematol..

[5] Karavasiloglou N., Hughes D.J., Murphy N., Schomburg L., Sun Q., Seher V., Rohrmann S., Weiderpass E., Tjønneland A., Olsen A. (2023). Prediagnostic serum calcium concentrations and risk of colorectal cancer development in 2 large European prospective cohorts. Am. J. Clin. Nutr..

[6] Jiang C., Zhou Q., Yi K., Yuan Y., Xie X. (2024). Colorectal cancer initiation: understanding early-stage disease for intervention. Cancer Lett..

[7] Fearon E.R. (2011). Molecular genetics of colorectal cancer. Annu. Rev. Pathol..

[8] Chapelle N., Martel M., Toes-Zoutendijk E., Barkun A.N., Bardou M. (2020). Recent advances in clinical practice: colorectal cancer chemoprevention in the average-risk population. Gut.

[9] Nikolouzakis T.K., Vassilopoulou L., Fragkiadaki P., Mariolis Sapsakos T., Papadakis G.Z., Spandidos D.A., Tsatsakis A.M., Tsiaoussis J. (2018). Improving diagnosis, prognosis and prediction by using biomarkers in CRC patients. Oncol. Rep..

[10] Shweikeh F., Zeng Y., Jabir A.R., Whittenberger E., Kadatane S.P., Huang Y., Mouchli M., Castillo D.R. (2024). The emerging role of blood-based biomarkers in early detection of colorectal cancer: a systematic review. Cancer Treat Res Commun.

[11] Hewitson P., Glasziou P., Watson E., Towler B., Irwig L. (2008). Cochrane systematic review of colorectal cancer screening using the fecal occult blood test (hemoccult): an update. Am. J. Gastroenterol..

[12] Brenner H., Stock C., Hoffmeister M. (2014). Effect of screening sigmoidoscopy and screening colonoscopy on colorectal cancer incidence and mortality: systematic review and meta-analysis of randomised controlled trials and observational studies. Bmj.

[13] Miller E.A., Pinsky P.F., Schoen R.E., Prorok P.C., Church T.R. (2019). Effect of flexible sigmoidoscopy screening on colorectal cancer incidence and mortality: long-term follow-up of the randomised US PLCO cancer screening trial. Lancet Gastroenterol. Hepatol..

[14] Gsur A., Baierl A., Brezina S. (2021). Colorectal cancer study of Austria (CORSA): a population-based multicenter study. Biology (Basel).

[15] Peters K.M., Carlson B.A., Gladyshev V.N., Tsuji P.A. (2018). Selenoproteins in colon cancer. Free Radic. Biol. Med..

[16] Short S.P., Pilat J.M., Williams C.S. (2018). Roles for selenium and selenoprotein P in the development, progression, and prevention of intestinal disease. Free Radic. Biol. Med..

[17] Alexander J., Olsen A.K. (2023). Selenium - a scoping review for nordic nutrition recommendations 2023. Food Nutr. Res., Suppl..

[18] Rayman M.P. (2008). Food-chain selenium and human health: emphasis on intake. Br. J. Nutr..

[19] Labunskyy V.M., Hatfield D.L., Gladyshev V.N. (2014). Selenoproteins: molecular pathways and physiological roles. Physiol. Rev..

[20] Hatfield D.L., Tsuji P.A., Carlson B.A., Gladyshev V.N. (2014). Selenium and selenocysteine: roles in cancer, health, and development. Trends Biochem. Sci..

[21] Hughes D.J., Fedirko V., Jenab M., Schomburg L., Méplan C., Freisling H., Bueno-de-Mesquita H.B., Hybsier S., Becker N.P., Czuban M. (2015). Selenium status is associated with colorectal cancer risk in the European prospective investigation of cancer and nutrition cohort. Int. J. Cancer.

[22] Cabral M., Kuxhaus O., Eichelmann F., Kopp J.F., Alker W., Hackler J., Kipp A.P., Schwerdtle T., Haase H., Schomburg L. (2021). Trace element profile and incidence of type 2 diabetes, cardiovascular disease and colorectal cancer: results from the EPIC-Potsdam cohort study. Eur. J. Nutr..

[23] Baker J.R., Umesh S., Jenab M., Schomburg L., Tjønneland A., Olsen A., Boutron-Ruault M.C., Rothwell J.A., Severi G., Katzke V. (2021). Prediagnostic blood selenium status and mortality among patients with colorectal cancer in western European populations. Biomedicines.

[24] Mishima E., Conrad M. (2022). Nutritional and metabolic control of ferroptosis. Annu. Rev. Nutr..

[25] Chen Z., Inague A., Kaushal K., Fazeli G., Schilling D., Xavier da Silva T.N., Dos Santos A.F., Cheytan T., Freitas F.P., Yildiz U. (2024). PRDX6 contributes to selenocysteine metabolism and ferroptosis resistance. Mol Cell.

[26] Ackermann T., Shokry E., Deshmukh R., Anand J., Galbraith L.C.A., Mitchell L., Rodriguez-Blanco G., Villar V.H., Sterken B.A., Nixon C. (2024). Breast cancer secretes anti-ferroptotic MUFAs and depends on selenoprotein synthesis for metastasis. EMBO Mol. Med..

[27] Moyad M.A. (2002). Selenium and vitamin E supplements for prostate cancer: evidence or embellishment?. Urology.

[28] Prabhu K.S. (2023). The selenoprotein P-LRP5/6-WNT3A complex promotes tumorigenesis in sporadic colorectal cancer. J. Clin. Investig..

[29] Demircan K., Bengtsson Y., Sun Q., Brange A., Vallon-Christersson J., Rijntjes E., Malmberg M., Saal L.H., Rydén L., Borg Å. (2021). Serum selenium, selenoprotein P and glutathione peroxidase 3 as predictors of mortality and recurrence following breast cancer diagnosis: a multicentre cohort study. Redox Biol..

[30] Demircan K., Jensen R.C., Chillon T.S., Jensen T.K., Sun Q., Bonnema S.J., Hackler J., Korevaar T.I.M., Glintborg D., Schomburg L. (2023). Serum selenium, selenoprotein P, and glutathione peroxidase 3 during early and late pregnancy in association with gestational diabetes mellitus: prospective Odense Child Cohort. Am. J. Clin. Nutr..

[31] Alexander J., Aaseth J.O., Schomburg L., Chillon T.S., Larsson A., Alehagen U. (2024). Circulating glutathione peroxidase-3 in elderly-association with renal function, cardiovascular mortality, and impact of selenium and coenzyme Q(10) supplementation. Antioxidants.

[32] Sun Q., Oltra E., Dijck-Brouwer D.A.J., Chillon T.S., Seemann P., Asaad S., Demircan K., Espejo-Oltra J.A., Sánchez-Fito T., Martín-Martínez E. (2023). Autoantibodies to selenoprotein P in chronic fatigue syndrome suggest selenium transport impairment and acquired resistance to thyroid hormone. Redox Biol..

[33] Demircan K., Sun Q., Bengtsson Y., Seemann P., Vallon-Christersson J., Malmberg M., Saal L.H., Rydén L., Minich W.B., Borg Å. (2022). Autoimmunity to selenoprotein P predicts breast cancer recurrence. Redox Biol..

[34] Kipp A.P. (2020). Selenium in colorectal and differentiated thyroid cancer. Hormones (Basel).

[35] Collaborators G.C.C. (2019). The global, regional, and national burden of colorectal cancer and its attributable risk factors in 195 countries and territories, 1990-2017: a systematic analysis for the Global Burden of Disease Study 2017. Lancet Gastroenterol. Hepatol..

[36] Schöttker B., Holleczek B., Hybsier S., Köhrle J., Schomburg L., Brenner H. (2024). Strong associations of serum selenoprotein P with all-cause mortality and mortality due to cancer, cardiovascular, respiratory and gastrointestinal diseases in older German adults. Eur. J. Epidemiol..

[37] Turan T.L., Klein H.J., Graf T.R., Chillon T.S., Plock J.A., Schomburg L. (2024). New-onset autoantibodies to selenoprotein P following severe burn injury. Front. Immunol..

[38] Vinceti M., Filippini T., Del Giovane C., Dennert G., Zwahlen M., Brinkman M., Zeegers M.P., Horneber M., D'Amico R., Crespi C.M. (2018). Selenium for preventing cancer. Cochrane Database Syst. Rev..

[39] Ala M., Kheyri Z. (2021). The rationale for selenium supplementation in inflammatory bowel disease: a mechanism-based point of view. Nutrition.

[40] Schomburg L. (2020). The other view: the trace element selenium as a micronutrient in thyroid disease, diabetes, and beyond. Hormones (Basel).

[41] Kipp A.P., Strohm D., Brigelius-Flohé R., Schomburg L., Bechthold A., Leschik-Bonnet E., Heseker H. (2015). Revised reference values for selenium intake. J. Trace Elem. Med. Biol..

[42] DeAngelo S.L., Győrffy B., Koutmos M., Shah Y.M. (2023). Selenoproteins and tRNA-Sec: regulators of cancer redox homeostasis. Trends Cancer.

[43] He L., Zhang L., Peng Y., He Z. (2024). Selenium in cancer management: exploring the therapeutic potential. Front. Oncol..

[44] Bertz M., Kühn K., Koeberle S.C., Müller M.F., Hoelzer D., Thies K., Deubel S., Thierbach R., Kipp A.P. (2018). Selenoprotein H controls cell cycle progression and proliferation of human colorectal cancer cells. Free Radic. Biol. Med..

[45] Speckmann B., Steinbrenner H. (2014). Selenium and selenoproteins in inflammatory bowel diseases and experimental colitis. Inflamm. Bowel Dis..

[46] Goltyaev M.V., Mal'tseva V.N., Varlamova E.G. (2020). Expression of ER-resident selenoproteins and activation of cancer cells apoptosis mechanisms under ER-stress conditions caused by methylseleninic acid. Gene (Amst.).

[47] Huang X., Yang X., Zhang M., Li T., Zhu K., Dong Y., Lei X., Yu Z., Lv C., Huang J. (2024). SELENOI functions as a key modulator of ferroptosis pathway in colitis and colorectal cancer. Adv. Sci. (Weinh.).

[48] Chen A., Huang H., Fang S., Hang Q. (2024). ROS: a "booster" for chronic inflammation and tumor metastasis. Biochim. Biophys. Acta Rev. Canc.

[49] Lupica-Tondo G.L., Arner E.N., Mogilenko D.A., Voss K. (2024). Immunometabolism of ferroptosis in the tumor microenvironment. Front. Oncol..

[50] Lee N., Kim D. (2025). Adapt or perish: efficient selenocysteine insertion is critical for metastasizing cancer cells. Cancer Res..

[51] Nascentes Melo L.M., Sabatier M., Ramesh V., Szylo K.J., Fraser C.S., Pon A., Mitchell E.C., Servage K.A., Allies G., Westedt I.V. (2025). Selenoprotein O promotes melanoma metastasis and regulates mitochondrial complex II activity. Cancer Res..

[52] Rua R.M., Nogales F., Carreras O., Ojeda M.L. (2023). Selenium, selenoproteins and cancer of the thyroid. J. Trace Elem. Med. Biol..

[53] Demircan K., Bengtsson Y., Chillon T.S., Vallon-Christersson J., Sun Q., Larsson C., Malmberg M., Saal L.H., Rydén L., Borg Å. (2023). Matched analysis of circulating selenium with the breast cancer selenotranscriptome: a multicentre prospective study. J. Transl. Med..

[54] Herrera-Abreu M.T., Guan J., Khalid U., Ning J., Costa M.R., Chan J., Li Q., Fortin J.P., Wong W.R., Perampalam P. (2024). Inhibition of GPX4 enhances CDK4/6 inhibitor and endocrine therapy activity in breast cancer. Nat. Commun..

[55] Barrett C.W., Reddy V.K., Short S.P., Motley A.K., Lintel M.K., Bradley A.M., Freeman T., Vallance J., Ning W., Parang B. (2015). Selenoprotein P influences colitis-induced tumorigenesis by mediating stemness and oxidative damage. J. Clin. Investig..

[56] Short S.P., Pilat J.M., Barrett C.W., Reddy V.K., Haberman Y., Hendren J.R., Marsh B.J., Keating C.E., Motley A.K., Hill K.E. (2021). Colonic epithelial-derived selenoprotein P is the source for antioxidant-mediated protection in colitis-associated cancer. Gastroenterology (New York, N. Y., 1943).

[57] McCann J.C., Ames B.N. (2011). Adaptive dysfunction of selenoproteins from the perspective of the triage theory: why modest selenium deficiency may increase risk of diseases of aging. FASEB J..

[58] Ramírez-Acosta S., Selma-Royo M., Collado M.C., Navarro-Roldán F., Abril N., García-Barrera T. (2022). Selenium supplementation influences mice testicular selenoproteins driven by gut microbiota. Sci. Rep..

[59] Kudva A.K., Shay A.E., Prabhu K.S. (2015). Selenium and inflammatory bowel disease. Am. J. Physiol. Gastrointest. Liver Physiol..

[60] Ferreira R.L.U., Sena-Evangelista K.C.M., de Azevedo E.P., Pinheiro F.I., Cobucci R.N., Pedrosa L.F.C. (2021). Selenium in human health and gut microflora: bioavailability of selenocompounds and relationship with diseases. Front. Nutr..

[61] Schomburg L. (2022). Selenoprotein P - selenium transport protein, enzyme and biomarker of selenium status. Free Radic. Biol. Med..

[62] Pilat J.M., Brown R.E., Chen Z., Berle N.J., Othon A.P., Washington M.K., Anant S.A., Kurokawa S., Ng V.H., Thompson J.J. (2023). SELENOP modifies sporadic colorectal carcinogenesis and WNT signaling activity through LRP5/6 interactions. J. Clin. Investig..

[63] Dreher I., Jakobs T.C., Köhrle J. (1997). Cloning and characterization of the human selenoprotein P promoter. Response of selenoprotein P expression to cytokines in liver cells. J. Biol. Chem..

[64] Renko K., Hofmann P.J., Stoedter M., Hollenbach B., Behrends T., Köhrle J., Schweizer U., Schomburg L. (2009). Down-regulation of the hepatic selenoprotein biosynthesis machinery impairs selenium metabolism during the acute phase response in mice. FASEB J..

[65] Pal A., Dhar A., Shamim M.A., Rani I., Negi R.R., Sharma A., Chatterjee N., Goyal A., Sadashiv, Kaur B. (2024). Selenium levels in colorectal cancer: a systematic review and meta-analysis of serum, plasma, and colorectal specimens. J. Trace Elem. Med. Biol..

[66] Mayne S.T., Ferrucci L.M., Cartmel B. (2012). Lessons learned from randomized clinical trials of micronutrient supplementation for cancer prevention. Annu. Rev. Nutr..

[67] Demircan K., Chillon T.S., Bang J., Gladyshev V.N., Schomburg L. (2024). Selenium, diabetes, and their intricate sex-specific relationship. Trends Endocrinol. Metabol..

[68] Kristal A.R., Darke A.K., Morris J.S., Tangen C.M., Goodman P.J., Thompson I.M., Meyskens F.L., Goodman G.E., Minasian L.M., Parnes H.L. (2014). Baseline selenium status and effects of selenium and vitamin e supplementation on prostate cancer risk. J. Natl. Cancer Inst..

[69] Geybels M.S., Verhage B.A., van Schooten F.J., Goldbohm R.A., van den Brandt P.A. (2013). Advanced prostate cancer risk in relation to toenail selenium levels. J. Natl. Cancer Inst..

[70] Alfthan G., Eurola M., Ekholm P., Venäläinen E.R., Root T., Korkalainen K., Hartikainen H., Salminen P., Hietaniemi V., Aspila P. (2015). Effects of nationwide addition of selenium to fertilizers on foods, and animal and human health in Finland: from deficiency to optimal selenium status of the population. J. Trace Elem. Med. Biol..

[71] Alehagen U., Aaseth J., Schomburg L., Larsson A., Opstad T., Alexander J. (2024). Selenoprotein P increases upon selenium and coenzyme Q(10) supplementation and is associated with telomere length, quality of life and reduced inflammation and mortality. Free Radic. Biol. Med..

[72] Alehagen U., Alexander J., Aaseth J. (2016). Supplementation with selenium and coenzyme Q10 reduces cardiovascular mortality in elderly with low selenium status. A secondary analysis of a randomised clinical trial. PLoS One.

