# Supplementary material for: Colorectal cancer mortality is associated with low selenoprotein P status at diagnosis
Source: Redox Biol. 2025 May 24;84:103701. doi: 10.1016/j.redox.2025.103701 (PMC12158609; doi:10.1016/j.redox.2025.103701)
Supplement: Multimedia component 1 [file mmc1.docx]

**SUPPLEMENTARY MATERIAL**

**Supplementary Table 1**. Association of selenium markers with death in all participants

|  | **Univariate** | | | **Fully adjusted*** | | |
| --- | --- | --- | --- | --- | --- | --- |
| **Characteristic** | **N** | **HR**^1^ | **95% CI**^1^ | **N** | **HR**^1^ | **95% CI**^1^ |
| **Selenium** (per SD increase) | 519 | 0.62 | 0.50, 0.76 | 519 | 0.84 | 0.69, 1.03 |
| **SELENOP** (per SD increase) | 519 | 0.56 | 0.49, 0.64 | 519 | 0.68 | 0.59, 0.78 |
| **GPx3 activity** (per SD increase) | 519 | 0.74 | 0.64, 0.85 | 519 | 0.86 | 0.74, 0.99 |
| ^1^HR; hazard ratio, CI; confidence interval, SD; standard deviation, CRC; colorectal cancer, Se; selenium, SELENOP; selenoprotein P, GPx3; glutathione peroxidase 3. *Adjustment for age, sex, BMI, smoking status, alcohol consumption, disease group.  **Supplementary Table 2.** Cluster characteristics   \| **Characteristic** \| **Cluster 1**  N = 130^1^ \| **Cluster 2**  N = 115^1^ \| **Cluster 3**  N = 274^1^ \| \| --- \| --- \| --- \| --- \| \| **Selenium (µg/L)** \| 81 (16) \| 49 (11) \| 66 (11) \| \| **SELENOP (mg/L)** \| 3.41 (0.71) \| 1.87 (0.74) \| 2.72 (0.69) \| \| **GPx3 activity (U/L)** \| 240 (41) \| 165 (50) \| 200 (35) \| \| ^1^Median (IQR), SELENOP; selenoprotein P, GPx3; glutathione peroxidase 3. \| \| \| \| | | | | | | |

**Supplementary Table 3.** Prevalence of SELENOP-aAb

| **Characteristic** | **Controls**  N = 153^1^ | **Adenoma**  N = 255^1^ | **CRC**  N = 111^1^ |
| --- | --- | --- | --- |
| **SELENOP aAb** |  |  |  |
| Negative | 152 (99%) | 241 (95%) | 105 (95%) |
| Positive | 1 (0.7%) | 14 (5.5%) | 6 (5.4%) |
| ^1^n (%), CRC; colorectal cancer, SELENOP; selenoprotein P, aAb; autoantibodies. | | | |


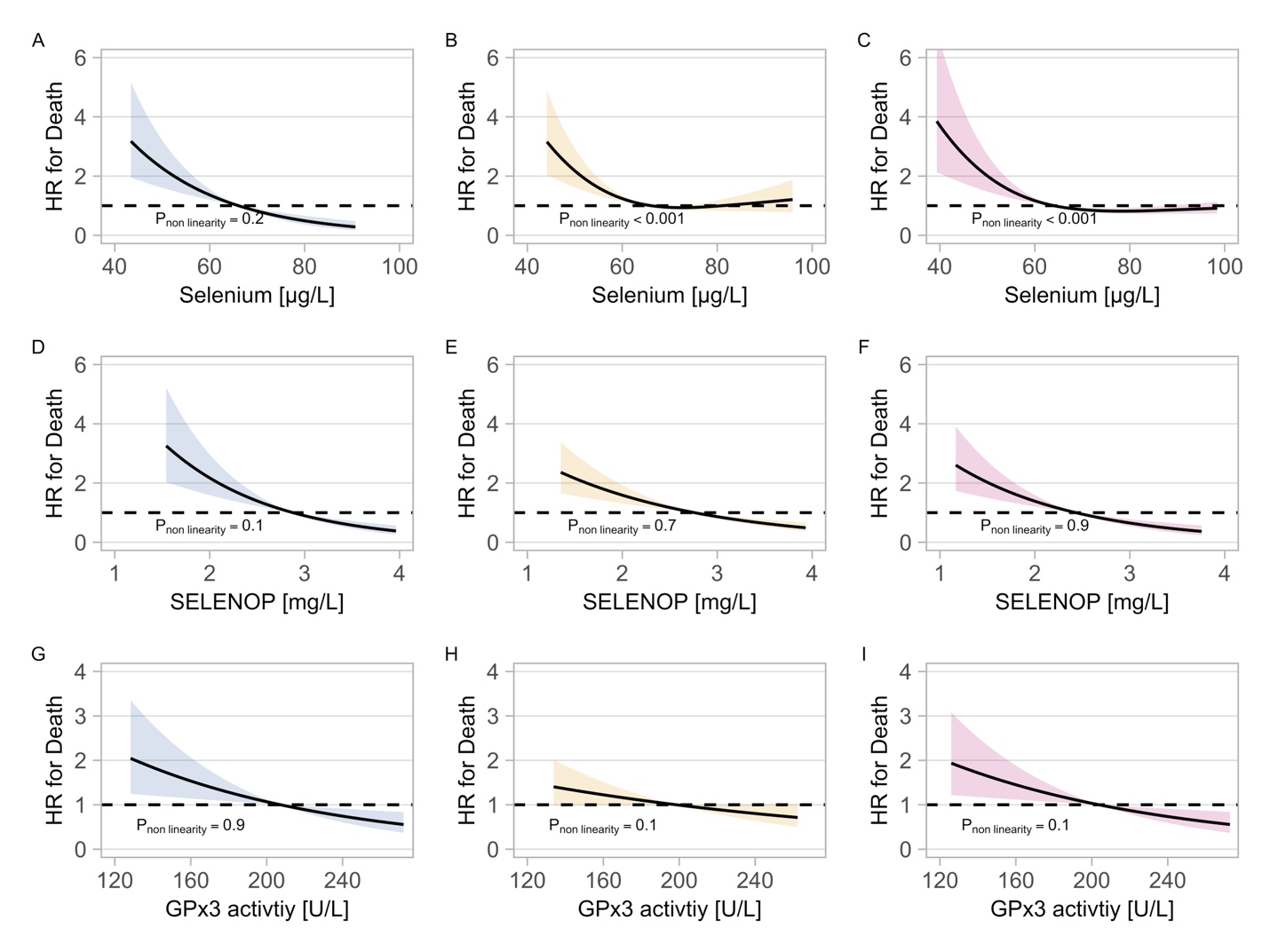


**Supplementary Figure 1.** Associations of selenium (Se), SELENOP and GPx3 with survival. Linear regression models with three knots at centiles 10, 50 and 90 were calculated to account for non-linearity. All models are univariate. Blue represents the control group, yellow the adenoma group, and red the colorectal cancer (CRC) group. **A) - C)** The association of Se with death in the different groups. **D) - F)** The association of SELENOP with death in the different groups. **G) - I)** The association of GPx3 activity with death in the different groups. HR; hazard ratio, SELENOP; selenoprotein P, GPx3; glutathione peroxidase 3.


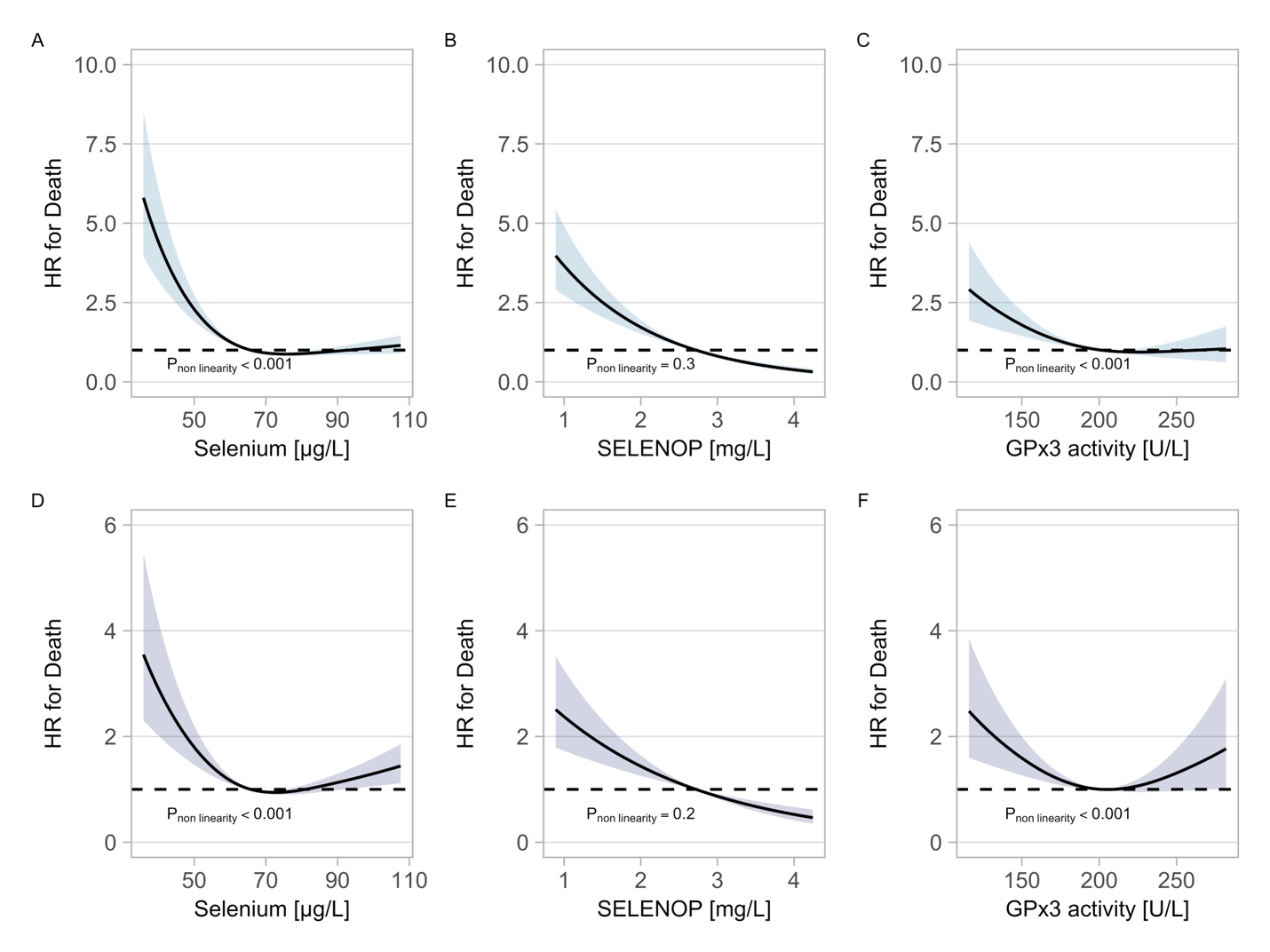


**Supplementary Figure 2.** Associations of selenium (Se), SELENOP, and GPx3 with survival. Linear regression models with 3 knots at centiles 10, 50 and 90 were calculated to account for non-linearity. The model is fully adjusted. Light blue shows the univariate model. Light purple shows the fully adjusted model. **A) - C)** Associations of the selenium marker with death with the univariate model. **D) - F)** Association of the selenium markers with death with the fully adjusted model. HR; hazard ratio, SELENOP; selenoprotein P, GPx3; glutathione peroxidase 3.
